# Human Intestinal Parasites

**Published:** 2007-12

**Authors:** Rashidul Haque

**Affiliations:** Laboratory Sciences Division, ICDDR,B, GPO Box 128, Dhaka 1000, Bangladesh Email: rhaque@icddrb.org

Parasitic infections, caused by intestinal helminths and protozoan parasites, are among the most prevalent infections in humans in developing countries. In developed countries, protozoan parasites more commonly cause gastrointestinal infections compared to helminths. Intestinal parasites cause a significant morbidity and mortality in endemic countries.

Helminths are worms with many cells. Nematodes (roundworms), cestodes (tapeworms), and trematodes (flatworms) are among the most common helminths that inhabit the human gut. Usually, helminths cannot multiply in the human body. Protozoan parasites that have only one cell can multiply inside the human body. There are four species of intestinal helminthic parasites, also known as geohelminths and soil-transmitted helminths: *Ascaris lumbricoides* (roundworm), *Trichiuris trichiuria* (whipworm), *Ancylostoma duodenale*, and *Necator americanicus* (hookworms). These infections are most prevalent in tropical and subtropical regions of the developing world where adequate water and sanitation facilities are lacking (1,2). Recent estimates suggest that *A. lumbricoides* can infect over a billion, *T. trichiura* 795 million, and hookworms 740 million people (3). Other species of intestinal helminths are not widely prevalent. Intestinal helminths rarely cause death. Instead, the burden of disease is related to less mortality than to the chronic and insidious effects on health and nutritional status of the host (4,5). In addition to their health effects, intestinal helminth infections also impair physical and mental growth of children, thwart educational achievement, and hinder economic development (6,7).

The most common intestinal protozoan parasites are: *Giardia intestinalis, Entamoeba histolytica, Cyclospora cayetanenensis*, and *Cryptosporidium* spp. The diseases caused by these intestinal protozoan parasites are known as giardiasis, amoebiasis, cyclosporiasis, and cryptosporidiosis respectively, and they are associated with diarrhoea (8). *G. intestinalis* is the most prevalent parasitic cause of diarrhoea in the developed world, and this infection is also very common in developing countries. Amoebiasis is the third leading cause of death from parasitic diseases worldwide, with its greatest impact on the people of developing countries. The World Health Organization (WHO) estimates that approximately 50 million people worldwide suffer from invasive amoebic infection each year, resulting in 40-100 thousand deaths annually (9,10). Cryptosporidiosis is becoming most prevalent in both developed and developing countries among patients with AIDS and among children aged less than five years. Several outbreaks of diarrhoeal disease caused by *C. cayetanensis* have been reported during the last decade (11). Spread of these protozoan parasites in developing countries mostly occurs through faecal contamination as a result of poor sewage and poor quality of water. Food and water-borne outbreaks of these protozoan parasites have occurred, and the infectious cyst form of the parasites is relatively resistant to chlorine (12). Other species of protozoan parasites can also be found in the human gut, but they are not pathogenic, except *Microsporidia* sp.

In an article published in this issue of the Journal, Jacobsen *et al.* looked at the prevalence of intestinal parasites in young Quichua children in the highland or rural Ecuador (13). They have found a high prevalence of intestinal parasites, especially the intestinal protozoan parasites. They have used the traditional microscopic technique to diagnose intestinal parasitic infections. In total, 203 stool samples were examined from children aged 12-60 months and found that 85.7% of them had at least on parasite. The overall prevalence of intestinal protozoan parasites were: *E. histolytica/E. dispar* 57.1%, *Escherichia coli* 34.0%, *G. intestinalis* 21.1%, *C. parvum* 8.9%, and *C. mesnili* 1.7%, while the prevalence of intestinal helminthic parasites in this study were: *A. lumbricoides* 35.5%, *T. trichiura* 0.5 %, *H. diminuta* 1.0%, and *S. stercoralis* 0.7%. A recent study in Nicaragua in asymptomatic individuals found that 12.1% (58/480) were positive for *E. histolytica/E. dispar* by microscopy, but *E. histolytica* and *E. disapr* were positive by polymerase chain reaction (PCR) only in three and four stool samples respectively among the microscopic positive samples (Unpublished data). This study proves again that the diagnosis of *E. histolytica/E. dispar* is neither sensitive nor specific when it is done by microscopy. To understand the real prevalence of *E. histolytica*-associated infection, a molecular method must be used for its diagnosis.

Over the last several years, we have seen new approaches to the diagnosis, treatment, and prevention of intestinal protozoan parasites. However, the diagnosis and treatment of intestinal helminth infections have not been changed much, and the traditional microscopic method can be used for their diagnosis. Antigen-detection tests are now commercially available for the diagnosis of all three major intestinal protozoan parasites. Diagnosis of *E. histolytica* cannot be done any longer by microscopy, since this parasite is morphologically similar to the non-pathogenic parasite *E. dispar*. *E. histolytica*-specific antigen-detection test is now commercially available from TechLab, Blacksburg, Virginia, for the detection of *E. histolytica* antigen in stool specimens (14,15). In several studies, this *E. histolytica*-specific antigen-detection test has been used for the specific detection of *E. histolytica* (16,17). These studies have found that this antigen-detection test is sensitive and specific for the detection of *E. histolytica*. In a study in Bangladesh, *E. histolytica*-specific antigen-detection test identified *E. histolytica* in 50 of 1,164 asymptomatic preschool children aged 2-5 years (18). In a study in Nicaragua among patients with diarrhoea, where *E. histolytica*-specific test has been used, found that the prevalence of *E. histolytica* was 0.5% (19). In a study conducted in a cohort of Bangladeshi children found that the prevalence of *E. histolytica* in diarrhoeal stool samples was 8.0% (20). No studies that have been carried till date using *E. histolytica*-specific diagnostic test reported the prevalence of *E. histolytica* more than 10%. In addition to the antigen-detection test, several PCR-based tests specific for *E. histolytica* have been developed and used for specific detection of *E. histolytica* (21,22). Rapid diagnostic test for the detection of *E. histolytica* antigen in stool specimens has also been reported (23).

Diagnosis of giardiasis is best accomplished by detection of *Giardia* antigen in stool, since the classic microscopic examination is less sensitive and specific. A recent comparison of nine different antigen-detection tests demonstrated that all had high sensitivity and specificity, except one (24). *Giardia*-specific antigen-detection tests are now also commercially available from several diagnostic companies, and their performance is quite good, except a few. In addition to antigen-detection tests, PCR-based test for the detection of *G. intestinalis* has also been reported (25). The population genetics of *Giardia* are complex. However, a recent genetic linkage study has confirmed the distinct grouping of *Giardia* in two major types (26). These two main genotypes/assemblages of *G. intestinals* are commonly known as: assemblage A and assemblage B of *G. intestinalis*. Differentiation of these two assemblages of *G. intestinalis* can only be done by PCR-based tests. Findings of the largest case-control study conducted to date on the relationship between genotypes of *G. intestinalis* and symptoms of patients have been published (27). This study has shown that the *Giardia* assemblage A infection is associated with diarrhoea. In contrast, *Giardia* assemblage B infection is significantly associated with asymptomatic *Giardia*-associated infection, which was found to occur at a significantly higher rate (18.0%) as detected by the antigen-detection test (27). The PCR-based approach allowed resolution of infection to the genotype level and brought some clarity to the findings of asymptomatic giardiasis. Similar large-scale case-control studies need to be carried out in other continents to understand more on the association of *Giardia* assemblages with diarrhoea/dysentery.

Diagnosis of cryptosporidiosis is also best accomplished by detection of *Cryptosporidium* spp. antigen in stool samples, since classic microscopic examination is less sensitive, and modified acid-fast staining is required. *Cryptosporidium* spp.-specific antigen-detection test has been used in several studies and has been found to be sensitive and specific compared to classic microscopic examination and PCR-based test (28,29). There are two main species of *Cryptosporidium* that infect humans: *C. hominis* (genotype I) and *C. parvum* (genotype II). The PCR-based test is required for differentiation of these two species of *Cryptosporidium* spp. (30). Both *C. hominis* and *C. parvum* have been found in humans. There are a few other species of *Cryptosporidium* that also can be found in humans (31–33). Rapid diagnostic tests for the detection of *G. lamblia* and *Cryptosporidium* spp. have also been reported (34,35). Multiplex PCR-based test for the detection of *E. histolytica*, *G. intestinalis*, and *Cryptosporidium* spp. has already been reported, and the development of multiplex antigen-detection test for these three common and pathogenic intestinal protozoan parasites is underway at TechLab, Blacksburg, Virginia (36, Herbain J. Personal communication, 2007). These modern antigen-detection tests and PCR-based tests need to be used for understanding the actual prevalence and epidemiology of these protozoan parasites.

Soil-transmitted helminth infections are invariably more prevalent in the poorest sections of the populations in endemic areas of developing countries. The goal is to reduce morbidity from soil-transmitted helminth infections to such levels that these infections are no longer of public-health importance. An additional goal is to improve the developmental, functional and intellectual capacity of affected children (37). Highly-effective, safe single-dose drugs, such as albendazole, now available, can be dispensed through healthcare services, school health programmes, and community interventions directed at vulnerable groups (38). As these infections are endemic in poor communities, more permanent control will only be feasible where chemotherapy is supplemented by improved water supplies and sanitation, strengthened by sanitation education. In the long term, this type of permanent transmission control will only be possible with improved living conditions through economic development. Intestinal protozoa multiply rapidly in their hosts, and as there is a lack of effective vaccines, chemotherapy has been the only practised way to treat individuals and reduce transmission. The current treatment modalities for intestinal protozoan parasites include metronidazole, iodoquinol, diloxanide furoate, paromomycin, chloroquine, and trimethoprim-sulphamethoxazole (39). Nitazoxanide, a broad-spectrum anti-parasitic agent, was reported to be better than placebo for the treatment of cryptosporidiosis in a double-blind study performed in Mexico (40). Genomes of these three important protozoan parasites have already been published (41–43), and studies are underway to understand protective immunity to these protozoan parasites to develop vaccines for them.
